# Embryonic Caffeine Exposure Acts via A1 Adenosine Receptors to Alter Adult Cardiac Function and DNA Methylation in Mice

**DOI:** 10.1371/journal.pone.0087547

**Published:** 2014-01-27

**Authors:** Daniela L. Buscariollo, Xiefan Fang, Victoria Greenwood, Huiling Xue, Scott A. Rivkees, Christopher C. Wendler

**Affiliations:** 1 Memorial Sloan-Kettering Cancer Center, New York City, New York, United States of America; 2 Department of Pediatrics, University of Florida College of Medicine, Gainesville, Florida, United States of America; 3 University of Connecticut, Storrs, Connecticut, United States of America; 4 Division of Genetics, Department of Medicine, Brigham and Women's Hospital, Harvard Medical School, Boston, Massachusetts, United States of America; Rutgers University -New Jersey Medical School, United States of America

## Abstract

Evidence indicates that disruption of normal prenatal development influences an individual's risk of developing obesity and cardiovascular disease as an adult. Thus, understanding how *in utero* exposure to chemical agents leads to increased susceptibility to adult diseases is a critical health related issue. Our aim was to determine whether adenosine A1 receptors (A1ARs) mediate the long-term effects of *in utero* caffeine exposure on cardiac function and whether these long-term effects are the result of changes in DNA methylation patterns in adult hearts. Pregnant A1AR knockout mice were treated with caffeine (20 mg/kg) or vehicle (0.09% NaCl) i.p. at embryonic day 8.5. This caffeine treatment results in serum levels equivalent to the consumption of 2–4 cups of coffee in humans. After dams gave birth, offspring were examined at 8–10 weeks of age. A1AR+/+ offspring treated *in utero* with caffeine were 10% heavier than vehicle controls. Using echocardiography, we observed altered cardiac function and morphology in adult mice exposed to caffeine *in utero*. Caffeine treatment decreased cardiac output by 11% and increased left ventricular wall thickness by 29% during diastole. Using DNA methylation arrays, we identified altered DNA methylation patterns in A1AR+/+ caffeine treated hearts, including 7719 differentially methylated regions (DMRs) within the genome and an overall decrease in DNA methylation of 26%. Analysis of genes associated with DMRs revealed that many are associated with cardiac hypertrophy. These data demonstrate that A1ARs mediate *in utero* caffeine effects on cardiac function and growth and that caffeine exposure leads to changes in DNA methylation.

## Introduction

Increasing evidence indicates that alteration of normal prenatal development influences an individual's lifetime risk of developing obesity and cardiovascular disease [1]–[6]. Thus, understanding how *in utero* exposure to chemical agents leads to increased susceptibility to adult diseases is an important issue.

One substance that fetuses are frequently exposed to is caffeine, a non-selective adenosine receptor antagonist. Caffeine consumption during the first month of pregnancy is reported by 60% of women, and 16% of pregnant mothers report consuming 150 mg or more per day [7]. Caffeine exerts many cellular effects, including influences on intracellular calcium levels and inhibition of phosphodiesterase; however, at serum concentrations observed with typical human consumption, the major effects of caffeine are due to a blockade of adenosine action at the level of adenosine receptors through competitive inhibition [8].

Adenosine levels increase dramatically under physiologically stressful conditions that include hypoxia, tissue ischemia, and inflammation [9]–[11]. Adenosine acts via cell surface G-protein coupled receptors, including A1, A2a, A2b, and A3 adenosine receptors [12]. Of these adenosine receptors, A1 adenosine receptors (A1ARs) have the highest affinity for adenosine and are the earliest expressed adenosine receptor subtype in the developing embryo [12], [13]. Showing how adenosine plays an important role in development, recent data indicate that a single dose of caffeine given to pregnant mice leads to reduced embryonic heart size and impaired cardiac function in adulthood [14]; however, the mechanisms by which these effects occur are not known.

At present, our understanding of the long-term effects of *in utero* caffeine exposure remains modest. In animal models, embryonic caffeine exposure leads to teratogenic effects, including ventricular septal defects and intrauterine growth retardation (IUGR) [14]–[19]. Other studies show that caffeine exposure as early as embryonic day 10.5 (E10.5) in mice can cause reduced embryonic cardiac tissue [14]. In addition, caffeine can induce defects in angiogenesis in zebrafish (*Danio rerio*) embryos [14], [20]. In mice and zebrafish, caffeine affects embryonic cardiac function by increasing heart rates [21], [22]. Caffeine exposure increases expression of the cardiac structural gene myosin heavy chain alpha (*Myh6*) in fetal rat hearts [23]. In humans, there is little evidence that fetal caffeine exposure leads to morphological defects, but prenatal caffeine exposure is associated with an increased risk of spontaneous abortions and reduced birth weight [24]–[31]. Studies examining the long-term consequences of *in utero* caffeine exposure in humans have not been performed.

One recognized mechanism for transmitting *in utero* stress into an increased risk of adult disease involves epigenetic changes that include altered DNA methylation, post-translational modifications of histone tails, and miRNA regulation [32], [33]. Changes in DNA methylation patterns occurring normally during early embryogenesis can be influenced by nutritional and environmental factors resulting in long lasting effects in adulthood [34]–[36].

To provide further insights into adenosine and caffeine action in the embryo, we assessed the role of A1ARs in transducing the embryonic effects of caffeine in the mouse model. We also assessed the effects of caffeine on epigenetic modifications specifically DNA methylation patterns, and finally we examined caffeine's long-term effects on the heart.

## Methods

### Ethics Statement

All animal experiments were approved by the Institutional Animal Care and Use Committee (IACUC) at Yale University. All animal research was conducted at Yale University and concluded before corresponding author moved to the University of Florida College of Medicine.

### Animals

Adenosine A1 receptor (A1AR) deficient mice were provided by Dr. Bertil Fredholm at the Karolinska Institutet in Stockholm, Sweden and were characterized [37]. These mice are on a mixed background (129/OlaHsd/C57BL) and breed normally with expected Mendelian frequency.

Timed matings were performed with A1AR+/− males and A1AR+/− females, and the day a vaginal plug was observed was designated as embryonic day 0.5 (E0.5). Pregnant dams were randomized into two groups and injected intraperitoneally (i.p.) at E8.5. This stage is a critical time during cardiac development when the heart has begun to function, the heart valves are forming, and the heart is beginning to loop in order to bring the different chambers of the heart into proper alignment [38], [39]. In addition, treatment of pregnant dams at E8.5 was chosen because it is during the embryonic development window (E6.5–10.5) when genomic DNA is being re-methylated [40], thus E8.5 is a stage sensitive to DNA methylation disruption. Group 1 was injected with vehicle 0.9% NaCl, and group 2 was injected with 20 mg/kg of caffeine (Sigma-Aldrich, St. Louis, MO, USA) dissolved in vehicle. This caffeine treatment results in circulating blood levels equivalent to the consumption of 2–4 cups of coffee in humans and 65% A1AR occupancy [8], [14]. Analysis was performed on male offspring divided into six groups based on treatment and genotype; including 1) vehicle/A1AR+/+ (veh+/+), 2) vehicle/A1AR+/− (veh+/−), 3) vehicle/A1AR−/− (veh−/−), 4) caffeine/A1AR+/+ (caff+/+), 5) caffeine/A1AR+/− (caff+/−), and 6) caffeine/A1AR−/− (caff−/−). Adult offspring for each group were obtained from at least 4 different dams. Adult offspring were used for nuclear magnetic resonance (NMR), weights, and echocardiography. The number of male offspring produced for these experiments came from 11 dams treated with vehicle including 8 A1AR+/+, 24 A1AR+/−, and 8 A1AR −/−, and 9 dams treated with caffeine including 8 A1AR+/+, 17 A1AR+/−, and 11 A1AR−/−. Of these mice some died before NMR and echocardiography, the Ns for each experiment are provided in the figure legends. In addition, 3–4 hearts from each group were used for histology and 3–4 hearts per group were used for RNA and DNA isolation. After birth, mice were weighed weekly from 2–8 weeks of age. Mice were euthanized by CO_2_ inhalation followed by cervical dislocation.

### Nuclear magnetic resonance

Between 8–10 weeks of age, the vehicle- and caffeine-treated male mice were evaluated by NMR at the Yale Metabolic Phenotyping Center, as described [14], [41]. Animals were placed in a restraint cylinder for body composition analysis using the Minispec Benchtop NMR (Bruker Optics, Billerica, MA, USA). NMR analysis was used to assess absolute fat, lean mass, and free body fluid content, based on total body weight. Data were used to determine percent body fat (fat mass/total body mass ×100).

### Echocardiography

Cardiac function of male offspring was assessed using echocardiography between 8–10 weeks of life, as described [14], [42]. Offspring were anesthetized with a continuous flow of isoflurane administered via nosecone and anesthesia levels were regulated to maintain heart rates between 400 and 500 beats per minute. Transthoracic 2D M-mode echocardiography was performed using a 30-MHz probe (Vevo 770; Visualsonics, Toronto, ON, Canada) [14]. Echocardiography and analysis of results were performed blinded.

The hemodynamic effects of caffeine treatment were assessed in dams as described [43]. Briefly, baseline data on heart rate and cardiac function were obtained as described above. Animals were allowed to recover for 30 minutes followed by treatment with either 0.9% NaCl (vehicle) or 20 mg/kg of caffeine. Echocardiography was then performed 30 minutes after treatment.

### Adult cardiac histology

Adult hearts of male offspring were fixed by perfusion of hearts with 4% paraformaldehyde solution (PFA; Electron Microscopy Sciences, Hatfield, PA, USA) containing 150 mM KCl and 5 mM EDTA. Hearts were embedded in paraffin, sectioned, mounted on slides, and analyzed as described [14].

### DNA methylation array analysis

Methylated DNA immunoprecipitation (MeDIP) and NimbleGen DNA methylation microarrays were used to assess changes in DNA methylation patterns between caffeine- and vehicle-treated adult male mice. The groups studied included: caff+/+, caff−/−, veh+/+, and veh−/− mice. Two samples from each treatment group were used to generate the DNA methylation array data, which was then used for all subsequent pathway analyses. The use of 1 to 2 samples per group is common for this type of analysis, so our use of two samples is consistent with previously published reports [44], [45]. Genomic DNA from left ventricles was isolated using the DNeasy Blood & Tissue Kit (Qiagen, Valencia, CA, USA). MeDIP was performed by following the NimbleGen DNA Methylation Microarrays Sample Preparation Instructions. Both MeDIP-enriched and non-enriched DNA were amplified using the GenomePlex Complete Whole Genome Amplification Kit (Sigma-Aldrich). Amplified DNA was hybridized to the Mouse DNA Methylation 2.1 M Deluxe Promoter Arrays (Roche NimbleGen, Madison, WI, USA) according to manufacturer's protocol.

DNA methylation array data were analyzed using methods developed by Palmke et al. 2011 [44] and by Tobias Straub (http://www.protocol-online.org/cgi-bin/prot/view_cache.cgi?ID=3973). Data were normalized within (Lowess-based) and between (quantile-based) arrays and probe level log2 ratio of Cy5/Cy3 (M-value) was used as a measure of MeDIP enrichment [46]. All DNA methylation array data were uploaded to the Gene Expression Omnibus (GEO) and can be accessed via http://www.ncbi.nlm.nih.gov/geo/; accession number GSE43030. Chromosomal distribution of DMR regions were analyzed by using Enrichment on Chromosome and Annotation (CEAS) in Galaxy/Cistrome [47]. Venn diagrams were constructed with Galaxy/Cistrome [47].

Pathway analysis of the genes associated with the DMRs was conducted using MetaCore Enrichment Analysis (Version 6.11, build 41105; GeneGo, Carlsbad, CA, USA) and Ingenuity Pathway Analysis (Ingenuity Systems, Redwood City, CA, USA). The lists of differentially methylated genes and miRNAs were uploaded separately into the applications. MetaCore enrichment ontologies used included Pathway Maps, Map Folders, Process Networks, Diseases (by Biomarkers), and Disease Biomarker Networks. Ingenuity ontologies analyzed included IPA Core Analysis and IPA-Tox.

Bisulfite sequencing (BS-seq) was used to analyze the DMRs within the gene promoters and performed as described [48]. Bisulfite specific primers were designed with Methyl Primer Express v1.0 (Applied Biosystems, Carlsbad, CA, USA). Sequence data were analyzed with DNAstar (SeqMan, Madison, WI, USA). The CpG methylation percentage was calculated as (total number of methylated CpG)/(number of CpG sites in each gene × number of colonies sequenced).

### Global DNA methylation and hydroxymethylation

Global DNA methylation and hydroxymethylation of the left ventricular genomic DNA were measured with the MethylFlash Methylated DNA Quantification Kit and MethylFlash Hydroxymethylated DNA Quantification Kit (Colorimetric; Epigentek Group, Farmingdale, NY, USA) according to the manufacturer's protocols. The OD_450_ nm intensity of the colorimetric reaction was measured with Synergy HT Multi-Mode Microplate Reader (BioTek, Winooski, VT, USA). Each sample was measured in triplicate, and assays were repeated twice.

### Real-time PCR analysis

Total RNA from left ventricles of adult male offspring was extracted with RNeasy Plus Mini Kit (Qiagen), according to the manufacturer's protocol. cDNA was synthesized using iScript cDNA Synthesis Kit (Bio-Rad, Hercules, CA, USA). Primers were as follows: *Mef2c* forward: GATGCCATCAGTGAATCAAAGG; *Mef2c* reverse: GTTGAAATGGCTGATGGATATCC; *Tnnt2* forward: CTGAGACAGAGGAGGCCAAC; *Tnnt2* reverse: TTCTCGAAGTGAGCCTCCAT. For *Myh6* and *Myh7*, primers were designed and synthesized by SABiosciences (Qiagen). For *β-actin*, primers were designed and synthesized by RealTimePrimers.com (Elkins Park, PA, USA), *β-actin* forward: AAGAGCTATGAGCTGCCTGA, *β-actin* reverse: TACGGATGTCAACGTCACAC. Relative abundance of target genes to *β-actin* transcripts in the cDNA libraries was determined with SYBR®Green (Applied Biosystems) in a GeneAmp 7300 Real Time PCR System (Applied Biosystems). Each sample was measured in three separate reactions on the same plate. This assay was repeated three times. Amplification efficiencies of the target genes and *β-actin* primer pairs were tested to ensure that they were not statistically different. Differences in expression between the treatment groups were calculated with the 2^−ΔΔCT^ method. Statistical differences between treatments were determined on the linearized 2^−ΔCT^ values.

### Caffeine assay

Caffeine levels were measured in the serum of dams by ELISA assay (Neogen, Lexington, KY, USA), as described [14]. A1AR KO female mice were treated i.p. with 20 mg/kg of caffeine and blood serum was collected 2 hours later.

### Statistical Analysis

Data are presented as means ± the standard error of the mean (SEM). Analysis was performed with the statistics software package included with Microsoft Excel (Microsoft, Redmond, WA, USA) and GraphPad Prism 6.0 (GraphPad Software Inc., La Jolla, CA, USA). Statistical comparisons between groups were performed with student's t-test assuming equal variance or with one-way or two-way ANOVA with Bonferroni's post-test comparison. P≤0.05 was considered to be statistically significant.

## Results

### Caffeine treatment has no effect on maternal cardiac function

Female A1AR+/+ mice treated with a caffeine dose of 20 mg/kg were analyzed for serum caffeine levels and cardiac function. This dose of caffeine results in a circulating serum caffeine level of 37.5±1.5 µM (N = 3), similar to that observed in C57Bl/6 mice [14]. To test if caffeine treatment alters the hemodynamics of treated dams, we measured the heart rates as beats per minute (bpm) and cardiac outputs as milliliters per minute (ml/min) of adult mice before and 30 minutes after treatment with either vehicle or 20 mg/kg of caffeine. There was no significant difference with caffeine treatment in heart rate from baseline (447±6 bpm) to 30 min after caffeine treatment (462±12.5 bpm, N = 4), or in cardiac output from baseline (14.8±1.6 ml/min) to 30 min after caffeine treatment (15.0±0.6 ml/min, N = 4). As a control dams were treated with vehicle (0.9% NaCl), and no significant differences were observed in heart rate between baseline (442±18 bpm) and 30 min after vehicle (478±8 bpm, N = 4)) or in cardiac output from baseline (14.7±0.6 ml/min) to 30 min after vehicle (15.7±1.7 ml/min, N = 4). In addition, no significant differences were observed between either baseline or peak heart rate or cardiac output when comparing vehicle to caffeine treated mice.

### *In utero* caffeine treatment leads to higher body weight in adult male mice

**Figure 1:**
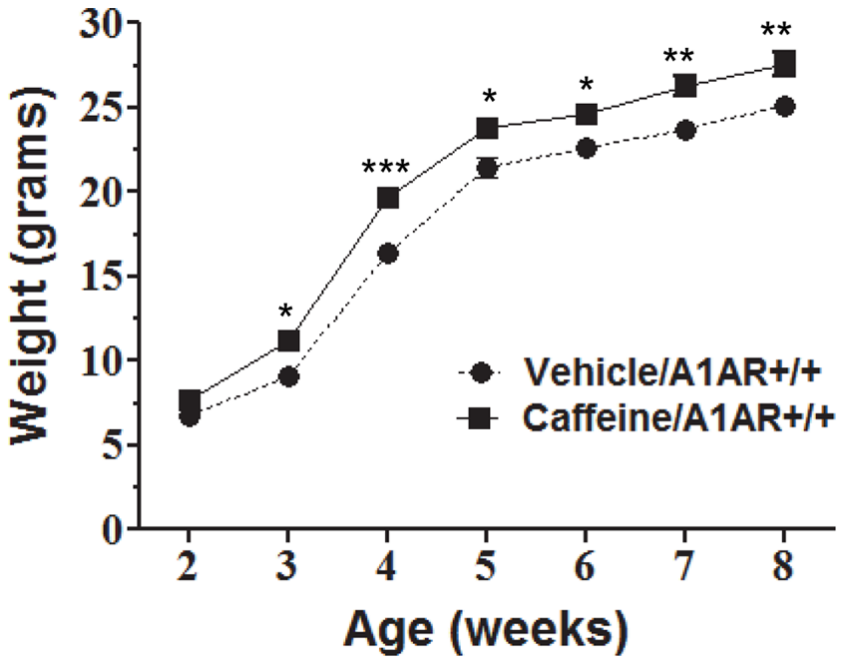
Embryonic caffeine exposure leads to increased weight in adulthood. Male mice treated *in utero* with caffeine were weighed every week starting 2 weeks after birth. Between 3–8 weeks of age, caffeine/A1AR+/+ mice were significantly heavier than vehicle/A1AR+/+ mice. Two-way ANOVA with Bonferroni post-test comparison was performed. *P≤0.05, **P≤0.01, ***P≤0.001. N = 8.

Pregnant dams were treated with one 20 mg/kg dose of caffeine or vehicle (0.9% NaCl) at E8.5. Male offspring were weighed weekly until 8 weeks of age. Beginning at 3 weeks of age, the caff+/+ mice were heavier than the veh+/+ controls (Fig. 1). The increase in body weight persisted throughout the study, caff+/+ mice weighed on average 2.47 grams more than veh+/+ controls. Because the absolute difference in body weight between the two groups was constant throughout the study, the percent increase in body weight peaked at 3 weeks with caff+/+ mice weighing 23.9% more than veh+/+ controls, and by 8 weeks of age the caff+/+ mice were 10% heavier than veh+/+ mice (Fig.1). Only A1AR+/+ mice treated with caffeine were significantly heavier in adulthood compared to A1AR+/+ controls. Comparisons of body weights between veh+/− (N = 20) and caff+/− (N = 15) or between veh−/− (N = 6) and caff−/− (N = 10) were not significantly different.

In addition to assessing body weight, we analyzed body fat in adult offspring by NMR. Even though there was a significant difference in body weight between the caffeine-treated group and the vehicle-treated group at the time NMRs were performed (two-way ANOVA, P≤0.04), no differences in the body fat content were detected among the different groups. The average percent body fat for the different treatment groups were veh+/+ 6.81±1.1% (N = 8), veh+/− 6.31±0.5% (N = 20), veh−/− 6.7±0.9% (N = 6), caff+/+ 6.96±0.8% (N = 8), caff+/− 6.22±0.8% (N = 15), and caff−/− 7.25±0.8% (N = 10). There was no difference in the percent muscle weight among the caffeine- and vehicle-treated groups (data not shown).

### *In utero* caffeine treatment causes a thickening of the left ventricular walls and altered cardiac function in adult hearts

**Figure 2:**
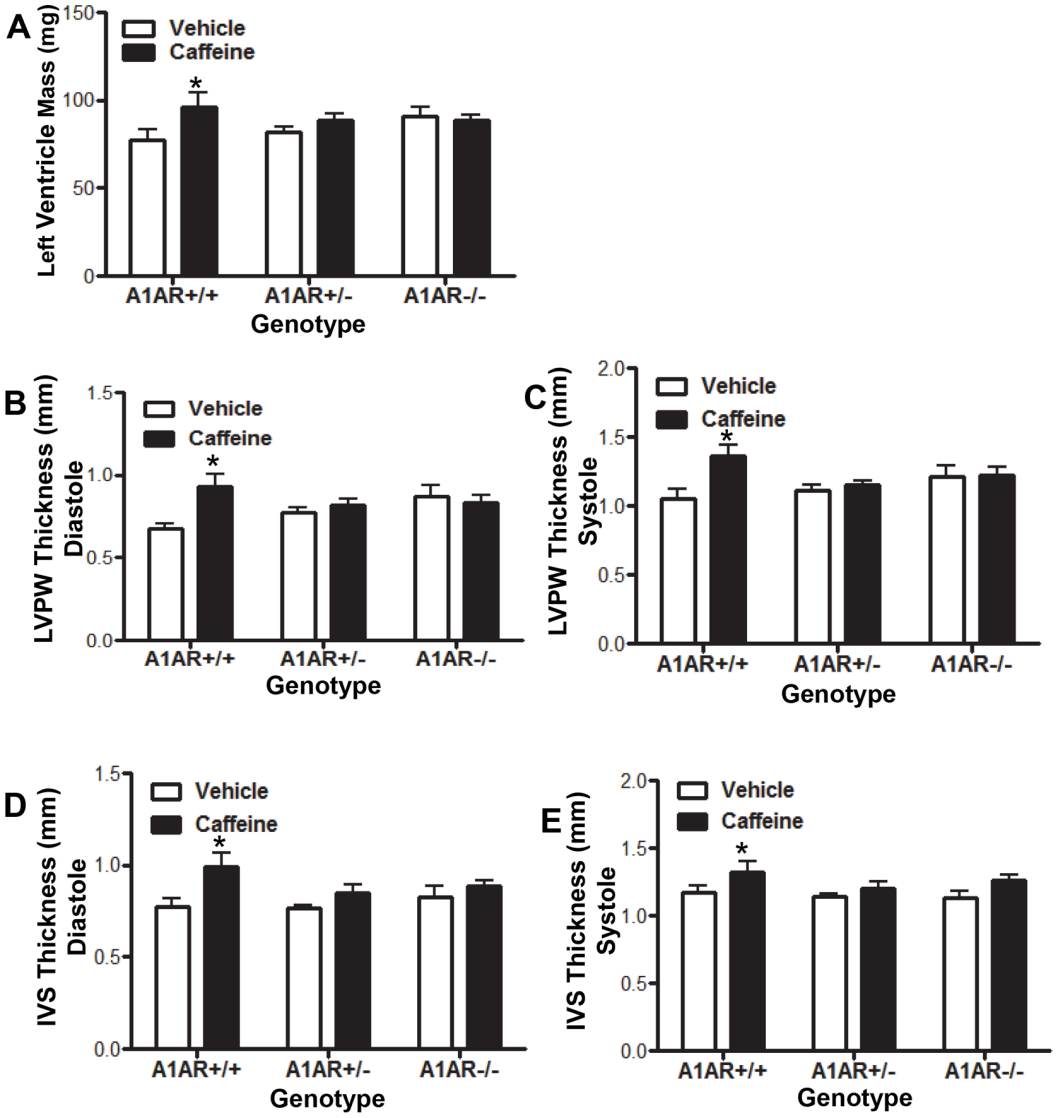
Embryonic caffeine exposure leads to thickening of left ventricular walls. Cardiac morphology was analyzed in adult mice by echocardiography at 8–10 weeks of age. (A) Caffeine-treated A1AR+/+ left ventricles were heavier than vehicle treated controls. Caffeine treatment of A1AR+/+ mice also caused increased thickness of the left ventricular posterior wall (LVPW) in both diastole (B) and systole (C), and increased thickness of the interventricular septum (IVS) in both diastole (D) and systole (E) when compared to vehicle controls. Vehicle/A1AR+/+, N = 6; Vehicle/A1AR +/−, N = 23; Vehicle/A1AR −/−, N = 8; caffeine/A1AR+/+, N = 6, caffeine/A1AR+/−, N = 15; caffeine/A1AR−/−, N = 10. Two-way ANOVA with Bonferroni post-test comparison was performed. *P≤0.05.

Cardiac function, wall thickness, and chamber size were measured by echocardiography in the adult male offspring of pregnant dams treated with caffeine or vehicle at E8.5. The groups examined included caffeine- or vehicle-treated and three genotypes (A1AR+/+, A1AR+/−, and A1AR−/−). Of these groups, only the caff+/+ vs. veh+/+ comparison revealed significant differences in cardiac function and morphology. Caffeine treatment lead to changes in adult cardiac morphology in the caff+/+ mice, including a 24% increase in left ventricle (LV) mass compared to veh+/+ (Fig. 2A). In addition, caffeine caused an increase in the thickness of both the left ventricular posterior wall (LVPW) and the interventricular septum (IVS; Fig. 2). The LVPW thickness was increased by 28.6% during diastole and 23.3% during systole, whereas the IVS thickness was increased by 24.5% during diastole and 14.3% during systole (Fig. 2).

**Figure 3:**
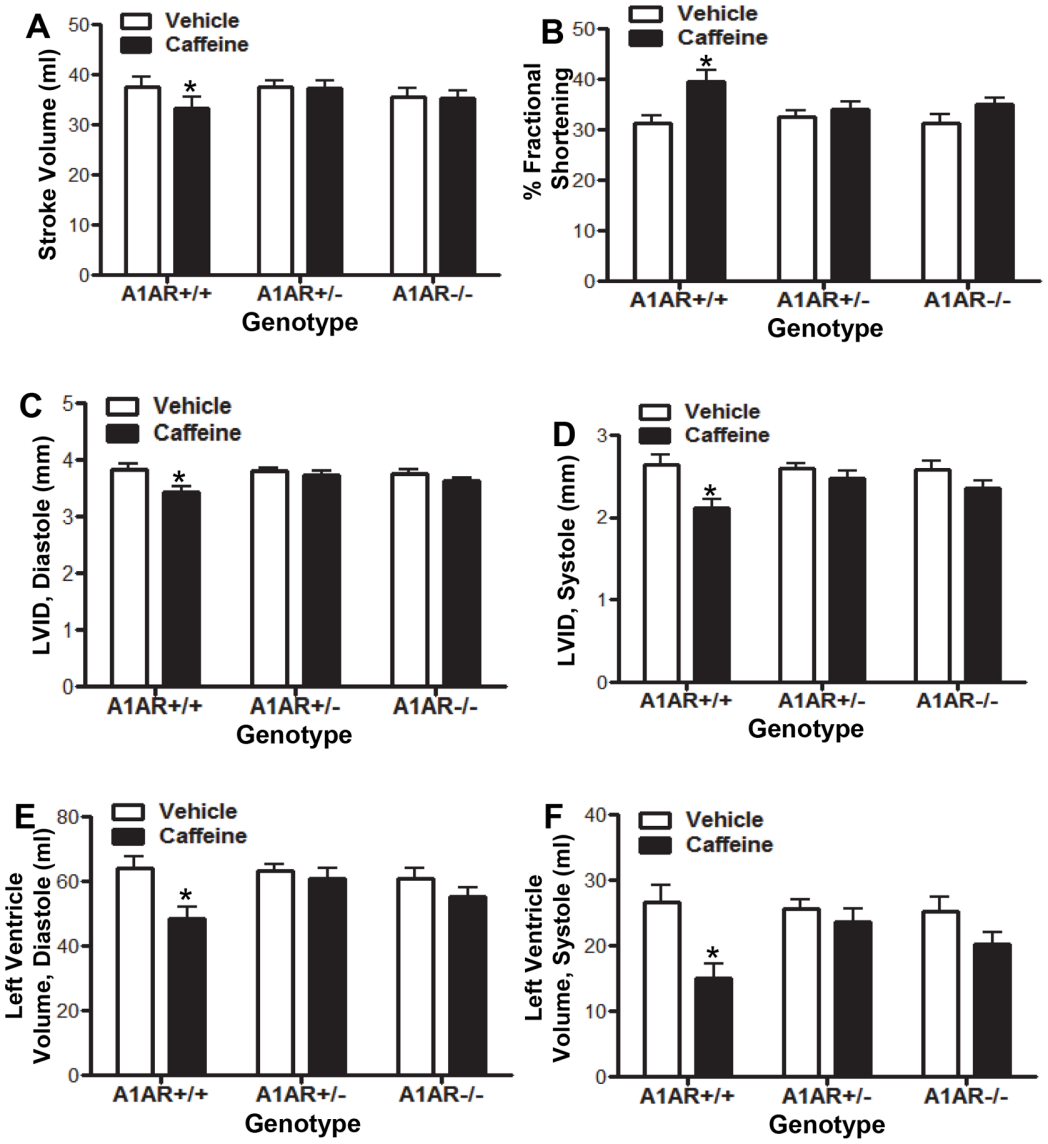
Embryonic caffeine exposure leads to altered cardiac function. Cardiac function of mice exposed to *in utero* caffeine was analyzed by echocardiography between 8–10 weeks of age. Analysis revealed that A1AR+/+ caffeine-treated mice had reduced stroke volume (A), increased % fractional shortening (B), reduced left ventricular internal diameter (LVID) in both diastole (C) and systole (D), and reduced left ventricle volume at both diastole (E) and systole (F). Vehicle/A1AR+/+, N = 6; Vehicle/A1AR+/−, N = 23; Vehicle/A1AR−/−, N = 8; caffeine/A1AR+/+, N = 6, caffeine/A1AR+/−, N = 15; caffeine/A1AR−/−, N = 10. Two-way ANOVA with Bonferroni post-test comparison was performed. *P≤0.05.

The increased left ventricular wall thickness was associated with a decrease in the left ventricular internal diameter (LVID) by 13.2% at diastole and 28.9% at systole (Fig. 3). The reduced LVID was associated with reduced left ventricle volume, which led to a 12.5% decrease in the LV stroke volume (Fig. 3). The percent fractional shorting (%FS) was increased in caffeine treated hearts (Fig. 3), and cardiac output (CO) was reduced by 11.4% in caff+/+ (N = 6) mice compared to veh+/+ (N = 6) treated mice (P≤0.02 student t-test). Although we observed differences in the cardiac output for the caff+/+ group, overt heart failure was not observed.

**Figure 4:**
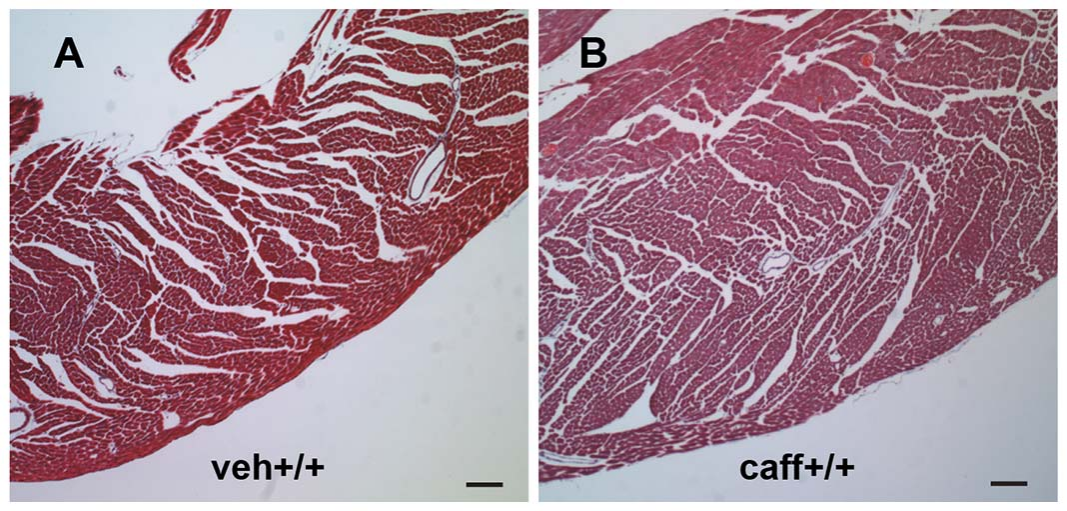
No effect on connective tissue deposition in adult hearts was observed with caffeine treatment. Adult hearts from mice exposed to *in utero* caffeine examined with trichrome stain to reveal heart muscle structure and connective tissue deposition. No differences in the amount of connective tissue deposition were observed between (A) veh+/+ and (B) caff+/+ adult left ventricles. However, caff+/+ left ventricles were thicker than veh+/+. N = 3. Scale bars = 100 µM.

Histological examination did not reveal differences in heart muscle structure among any of the groups but the caff+/+ group displayed thicker left ventricular walls compared to veh+/+ controls (Fig. 4). Trichrome staining indicated that there were no differences in connective tissue deposition or any evidence of scarring in adult hearts from any of the treatment groups (Fig.4).

### *In utero* caffeine exposure alters the DNA methylation pattern in adult hearts

NimbleGen DNA methylation microarrays were used to investigate DNA methylation patterns in adult left ventricles. The Mouse DNA Methylation 2.1 M Deluxe Promoter Array was chosen because it interrogates DNA methylation in 599 miRNA promoters including 15 kb upstream of the transcriptional start site (TSS), 15,969 gene promoters regions that range from 8,000 bp upstream to 3,000 bp downstream of the TSS, and 24,507 known CpG islands in the genomic DNA. For the DNA methylation analysis, four groups were studied including veh+/+, veh−/−, caff+/+, and caff−/− groups. This analysis identified changes in DNA methylation patterns that were caffeine- and A1AR-dependent (veh+/+ vs. caff+/+), caffeine-dependent and A1AR-independent (veh−/− vs. caff−/−), and A1AR-dependent and caffeine-independent (veh+/+ vs. veh−/−).

**Figure 5:**
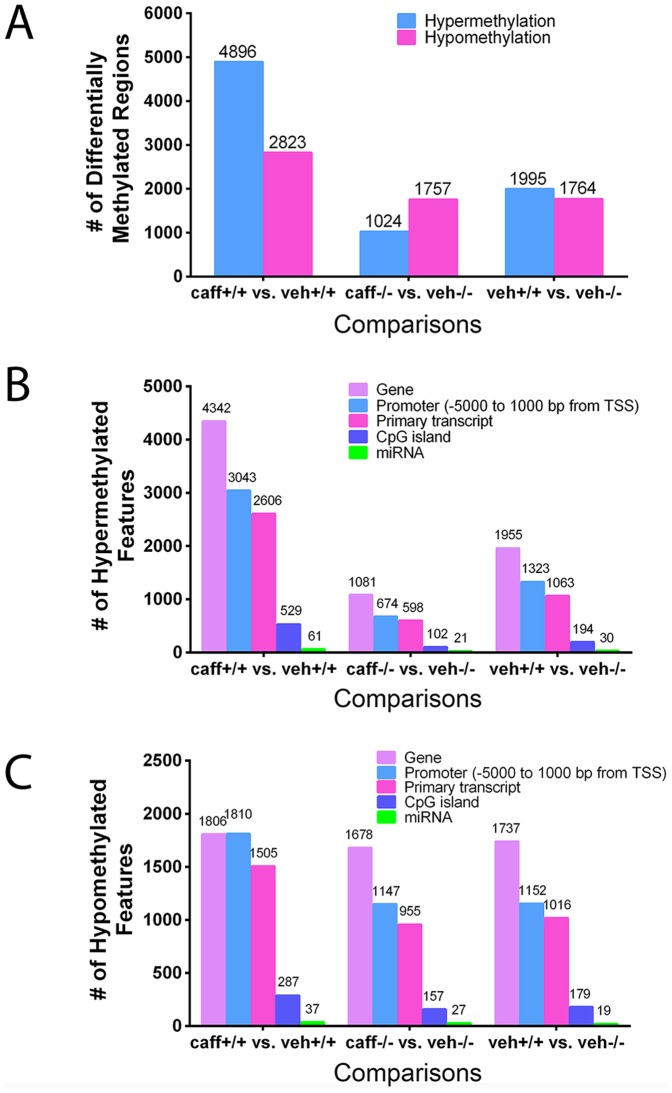
Embryonic caffeine exposure caused a change in DNA methylation patterns. Vehicle and caffeine treated mice with the same genotype were compared to identify differentially methylated regions (DMRs) in the genome (A). The veh+/+ vs. caff+/+ comparison identified the most differentially methylated regions in the genome following *in utero* caffeine exposure. Both (B) hypermethylated and (C) hypomethylated DMRs were detected throughout the genome with each comparison. Most DMRs were detected within the gene or promoter regions of the genome. The number of DNA samples per treatment group analyzed was 2.

Analysis of the different groups revealed that the veh+/+ vs. caff+/+ comparison had the greatest number of differentially methylated regions (DMRs) within the genomic DNA including both hypermethylated regions (4896) and hypomethylated regions (2823; Fig. 5A). Caffeine altered the DNA methylation pattern in the absence of A1AR expression (A1AR−/− mice) to a lesser degree than in A1AR+/+ mice. For example, the comparison of veh−/− vs. caff−/− only had 1024 hypermethylated regions and 1757 hypomethylated regions (Fig. 5A). In addition, the loss of A1AR expression alone altered DNA methylation patterns (Fig. 5A). Analysis of the differentially methylated regions revealed where in the genome the DMRs (either hypermethylated or hypomethylated) were located, including promoter regions, primary transcripts, known CpG islands or miRNA promoter regions (Fig. 5B, C).

**Figure 6:**
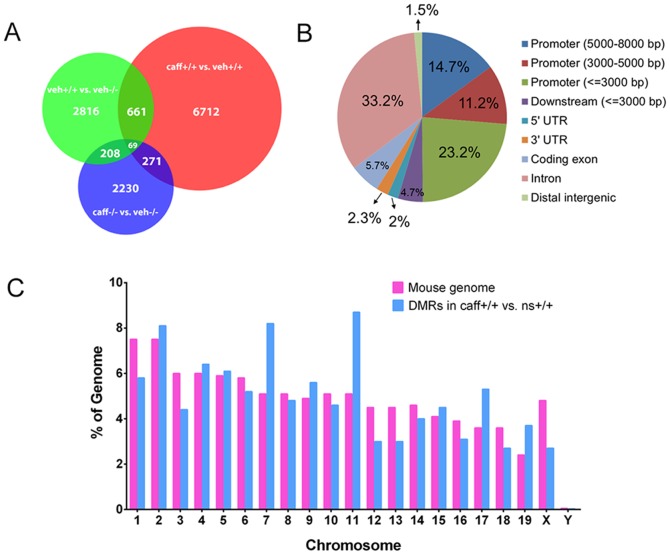
Distribution of the differentially methylated regions. (A) A Venn diagram indicates the number of DMRs that are shared by different comparison groups. The majority of DMRs within a comparison group are unique to that group with few regions detected in multiple comparison groups, and only 69 DMRs present in all three comparisons. (B) This chart illustrates locations in the genome for the DMRs identified from the veh+/+ vs. caff+/+ comparison. Analysis demonstrates that promoter regions from −1 to −3000 and intron regions contain the greatest percentage of DMRs. (C) In this chart, pink bars represent the percent of the genome that each chromosome contains and the blue bars are the percent of the total number of DMRs that are located on each chromosome. This chart identifies chromosomes 2, 7, and 11 as having the highest percentage of DMRs. N = 2.

A Venn diagram demonstrating the number of over-lapping DMRs from the different comparison groups indicated that the majority of DMRs are specific to each of the comparison groups (Fig. 6). The majority of DMRs in the veh+/+ vs. caff+/+ group were located in promoter regions less than 3,000 bp from the transcriptional start site (TSS) and in introns (Fig. 6). Further analysis of veh+/+ vs. caff+/+ identified the percentage of the total number of DMRs that were located on each chromosome (Fig. 6). The highest percentages of DMRs were located on chromosomes 2, 7 and 11, while the lowest percentage of DMRs was found on chromosome Y (Fig. 6).

### Caffeine treatment alters DNA methylation of genes associated with cardiac hypertrophy

Because the veh+/+ vs. caff+/+ comparison revealed phenotypic differences in cardiac function and the highest degree of DNA methylation differences, all 7719 DMRs identified from this comparison, both hyper- and hypomethylated regions, were used for gene pathway analysis. Genes associated with these DMRs were examined with the functional ontology enrichment tool from MetaCore. MetaCore uses different manually created groupings of genes from different databases, including common cellular processes, networks, biological function, and disease, which are referred to as ontologies. These ontologies are used to identify gene pathways that contain genes associated with differentially methylated regions of the genome.

**Figure 7:**
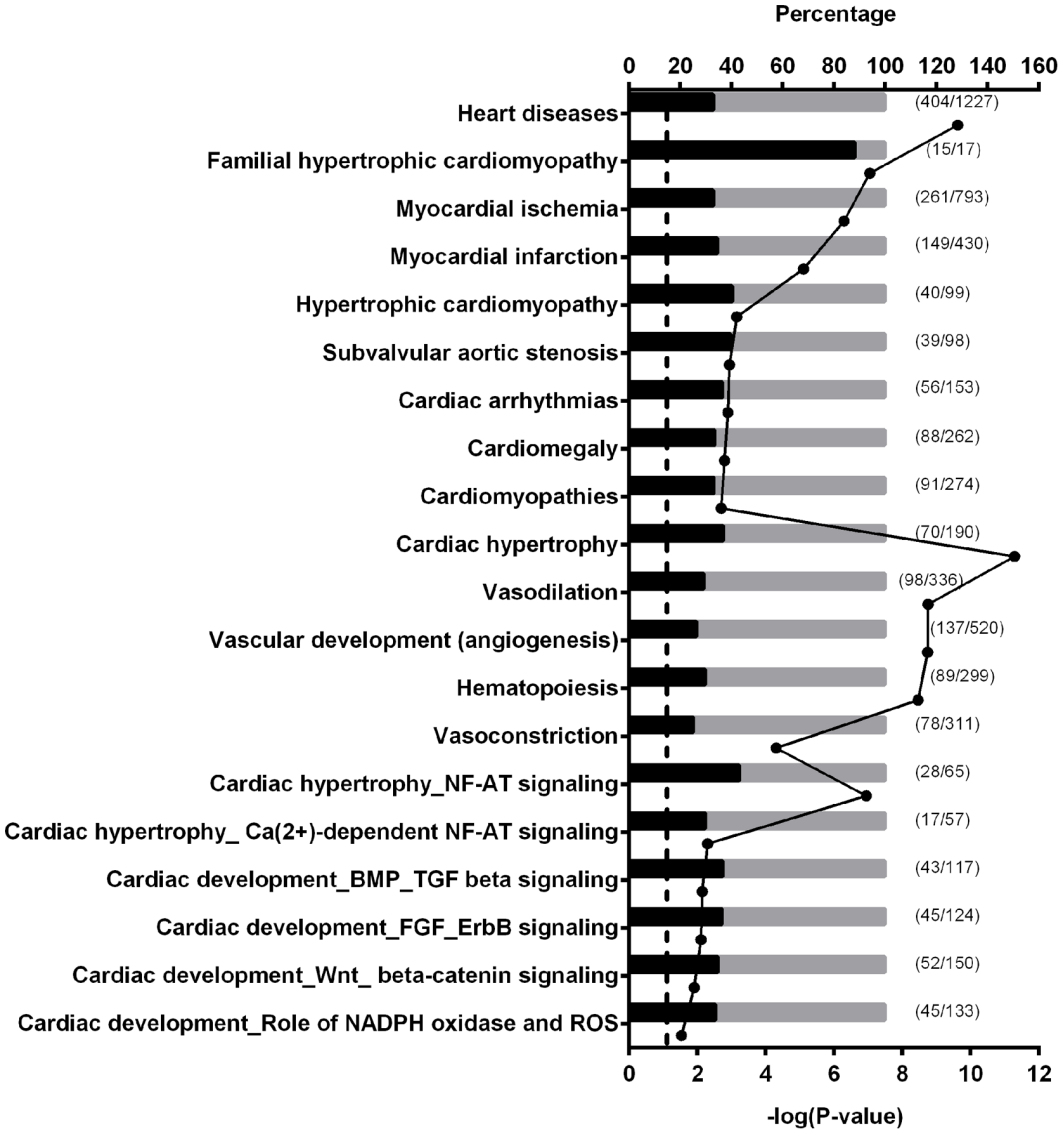
Significantly enriched cardiovascular related pathways. Gene set enrichment analysis was done with the differentially methylated genes between A1AR+/+ mice treated with or without caffeine. The analysis was conducted with MetaCore Enrichment Analysis using the ontologies of Diseases (by Biomarkers), Map Folders, Pathway Maps, and Process Networks. Bars represent the percentage of altered methylation genes (in black) within a pathway. The numbers of altered genes and genes in a pathway are listed next to the bars, which represent the percentage of altered genes within a pathway. Dots indicate the negative log 10 of the P-values. Larger –log(P-value) means that the pathway is more significant. The threshold for significance is marked in the graph as a dotted-line at 1.3 (−log(0.05)). N = 2.

The first ontology examined was “Pathway Maps,” which groups genes into cellular processes, protein functions, and diseases. The top 20 most significantly enriched pathways within the Pathway Maps ontology included cytoskeleton remodeling, G-protein signaling, and NF-AT signaling in cardiac hypertrophy (Fig. 7, Table 1). Next, Map Folder ontology, which is a higher order analysis of the Pathway Maps ontology, was applied. Map Folder ontology group genes together from the Pathway Maps database according to main biological processes. The top pathways identified by the Map Folder ontology included cell differentiation, cardiac hypertrophy, and vascular development (Fig. 7, Table 1).

**Table 1:** Top 20 most significantly enriched pathways analyzed with MetaCore™ontology (N = 2).

	#	Pathways	p-value	# of changed genes	# of genes in pathway
**Pathway map folders**	1	Cell differentiation	1.740e-23	264	928
	2	Inflammatory response	4.068e-22	215	715
	3	Tissue remodeling and wound repair	2.392e-19	168	534
	4	Immune system response	2.051e-15	249	973
	5	DNA-damage response	4.809e-14	114	354
	6	Cell cycle and its regulation	6.934e-14	134	444
	7	Apoptosis	1.836e-13	217	846
	8	Mitogenic signaling	1.894e-13	158	560
	9	Calcium signaling	1.913e-12	127	430
	10	Cardiac hypertrophy	5.033e-12	70	190
	11	Vasodilation	1.730e-9	98	336
	12	Vascular development (angiogenesis)	1.790e-9	137	520
	13	Hematopoiesis	3.383e-9	89	299
	14	Cystic fibrosis disease	7.457e-7	150	636
	15	Protein synthesis	7.583e-7	83	304
	16	Protein degradation	1.106e-6	75	269
	17	Vasoconstriction	4.797e-5	78	311
	18	Myogenesis regulation	1.054e-3	28	95
	19	Blood clotting	1.535e-3	65	278
	20	Transcription regulation	4.933e-3	8	18
**Pathway maps**	1	Cytoskeleton remodeling_TGF, WNT and cytoskeletal remodeling	1.153E-11	47	111
	2	Cytoskeleton remodeling_Cytoskeleton remodeling	2.479E-11	44	102
	3	Cell adhesion_Chemokines and adhesion	7.871E-10	41	100
	4	Cytoskeleton remodeling_FAK signaling	3.013E-09	28	57
	5	Cytoskeleton remodeling_Regulation of actin cytoskeleton by Rho GTPases	9.497E-09	16	23
	6	Signal transduction_JNK pathway	3.362E-08	22	42
	7	Cytoskeleton remodeling_Role of PKA in cytoskeleton reorganisation	6.569E-08	21	40
	8	Signal transduction_Erk interactions: Inhibition of Erk	7.428E-08	19	34
	9	Cardiac hypertrophy_NF-AT signaling in cardiac hypertrophy	1.099E-07	28	65
	10	G-protein signaling_RAC1 in cellular process	1.388E-07	19	35
	11	Immune response _CCR3 signaling in eosinophils	1.513E-07	31	77
	12	Development_Thrombopoietin-regulated cell processes	1.675E-07	22	45
	13	Development_A2A receptor signaling	3.276E-07	21	43
	14	Development_HGF signaling pathway	4.398E-07	22	47
	15	G-protein signaling_G-Protein alpha-12 signaling pathway	4.410E-07	19	37
	16	Immune response_Gastrin in inflammatory response	4.963E-07	28	69
	17	Development_ERK5 in cell proliferation and neuronal survival	9.780E-07	14	23
	18	Immune response_HMGB1/RAGE signaling pathway	1.280E-06	23	53
	19	Development_Endothelin-1/EDNRA signaling	1.280E-06	23	53
	20	Cell cycle_Influence of Ras and Rho proteins on G1/S transition	1.280E-06	23	53

The Process Networks ontology uses data from Pathway Maps, GO-processes, and network models of main cellular processes to identify significant gene pathways. The top 20 pathways identified by Process Network ontology included those involved with cytoskeleton regulation, cardiac development - BMP/TGF-beta signaling, and cardiac development – FGF/ErbB signaling (Fig. 7, Table 2).

**Table 2:** Top 20 most significantly enriched pathways analyzed with MetaCore™ontology (N = 2).

	#	Pathways	p-value	# of changed genes	# of genes in pathway
**Disease pathways**	1	Psychiatry and psychology	2.383E-11	856	2837
	2	Mental disorders	5.433E-11	848	2817
	3	Heart diseases	2.347E-10	404	1227
	4	Pathological conditions, signs and symptoms	1.042E-08	1191	4182
	5	Familial hypertrophic cardiomyopathy	8.802E-08	15	17
	6	Pathologic processes	9.621E-08	789	2694
	7	Sensation disorders	3.025E-07	114	296
	8	Nervous system diseases	3.070E-07	1344	4828
	9	Myocardial ischemia	4.967E-07	261	793
	10	Signs and symptoms	6.613E-07	717	2453
	11	Functional colonic diseases	1.173E-06	23	36
	12	Irritable bowel syndrome	1.173E-06	23	36
	13	Anorexia	1.178E-06	32	58
	14	Tobacco use disorder	1.589E-06	116	311
	15	Renal insufficiency	2.090E-06	105	277
	16	Vesico-ureteral reflux	4.129E-06	16	22
	17	Short bowel syndrome	4.265E-06	9	9
	18	Myocardial infarction	7.675E-06	149	430
	19	Body weight changes	1.748E-05	67	167
	20	Urologic diseases	2.104E-05	457	1539
**Process networks**	1	Cytoskeleton_Regulation of cytoskeleton rearrangement	9.303E-11	88	183
	2	Cytoskeleton_Actin filaments	2.240E-05	71	176
	3	Muscle contraction	2.270E-05	70	173
	4	Cell adhesion_Cadherins	5.363E-05	71	180
	5	Cell adhesion_Leucocyte chemotaxis	5.398E-05	79	205
	6	Signal Transduction_Cholecystokinin signaling	7.924E-05	46	106
	7	Development_Neurogenesis_Axonal guidance	9.138E-05	86	230
	8	Development_ERK5 in cell proliferation and neuronal survival	1.878E-04	15	24
	9	Reproduction_Spermatogenesis, motility and copulation	6.576E-04	82	229
	10	Signal transduction_WNT signaling	1.103E-03	65	177
	11	Proliferation_Positive regulation cell proliferation	1.416E-03	78	221
	12	Cell adhesion_Platelet aggregation	3.250E-03	62	174
	13	Cell adhesion_Integrin-mediated cell-matrix adhesion	3.411E-03	74	214
	14	Reproduction_Male sex differentiation	4.308E-03	83	246
	15	Signal transduction_NOTCH signaling	4.369E-03	80	236
	16	Muscle contraction_Nitric oxide signaling in the cardiovascular system	5.285E-03	46	125
	17	Neurophysiological process_Transmission of nerve impulse	6.304E-03	72	212
	18	Cell cycle_Mitosis	6.696E-03	62	179
	19	Cardiac development_BMP_TGF beta signaling	7.037E-03	43	117
	20	Cardiac development_FGF_ErbB signaling	7.692E-03	45	124

**Figure 8:**
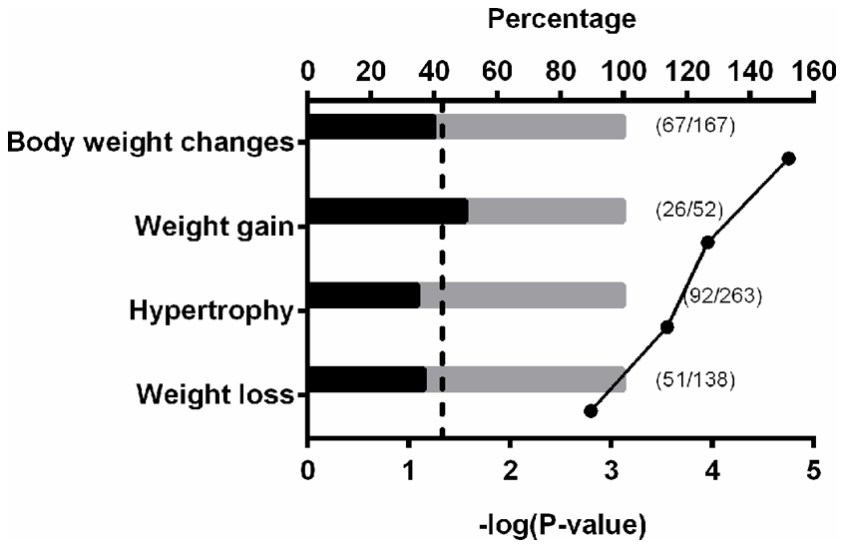
Significantly enriched body weight related pathways. Gene set enrichment analysis was performed with the differentially methylated genes between A1AR+/+ mice treated with or without caffeine. The analysis was conducted with MetaCore Enrichment Analysis using the ontologies of Diseases (by Biomarkers), Map Folders, Pathway Maps, and Process Networks. Bars represent the percentage of altered methylation genes (in black) within a pathway. The numbers of altered genes and genes in a pathway are listed next to the bars. Dots indicate the negative log 10 of the P-values. Larger –log(P-value) means that the pathway is more significant. The threshold for significance is marked in the graph as a dotted-line at 1.3 (−log(0.05)). N = 2.

The forth ontology examined, Disease (by Biomarker), uses biomarkers to group the genes into disease pathways. The Disease (by Biomarkers) ontology identified heart diseases, cardiomyopathy, myocardial ischemia, myocardial infarction, and body weight changes as some of the most significant pathways in this ontology analysis (Figs. 7–8, Table 2). There were many significantly enriched pathways related to the cardiovascular system including those related to cardiac disease, cardiac hypertrophy, and cardiac development and all these significant pathways are displayed together along with their P-values and the percentage of genes in the specific pathway that are affected (Fig. 7). Some pathways associated with growth were identified, but only four of these pathways had significant P-values (Fig. 8).

Further pathway analysis was performed by importing the genes associated with DMRs into the Ingenuity Pathway Analysis (IPA) software. This analysis identified cardiac specific pathways in several larger ontologies including Diseases and Disorders, Physiological Development, Top Toxicity Lists, and Cardiotoxicity (Table 3). Many cardiac pathways were identified with the Ingenuity database including Cardiovascular Disease, Organ Development, Cardiac Hypertrophy, and Cardiac Output (Table 3). Many of the pathways found with Ingenuity were similar to those identified with the MetaCore software (Table 1, Fig. 7).

**Table 3:** Significantly enriched miRNA pathways from Ingenuity Pathway Analysis following *in utero* caffeine exposure. (N = 2).

	Top pathways	p-value	# of changed genes
**Diseases and disorders**	Reproductive system disease	9.04E-17	18
	Cancer	1.61E-14	26
	Hematological disease	1.97E-12	10
	Endocrine system disorders	1.27E-11	16
	Inflammatory disease	1.03E-09	11
**Physiological development**	Connective tissue development and function	1.39E-03	4
	Nervous system development and function	2.22E-03	1
	Respiratory system development and function	2.22E-03	1
	Tissue development	2.22E-03	3
	Tumor morphology	2.22E-03	2
**Cardiotoxicity**	Cardiac inflammation	4.78E-06	2
	Congenital heart anomaly	2.60E-04	2
	Cardiac dilation	4.14E-02	1
	Cardiac infarction	4.51E-02	2
	Pulmonary hypertension	4.78E-02	1

Of the top 20 most significant cardiovascular pathways identified from the different ontologies in MetaCore, five were related to cardiac hypertrophy and four were related to cardiac development (Fig. 7). Further analysis, of the genes affected within the hypertrophic cardiomyopathy ontology (Fig. 7), identified many structural genes including troponin I (*Tnni3*), troponin T (*Tnnt2*), troponin C (*Tnnc1*), α-actin C1 (*Actc1*) that are important for proper cardiac function (Table 4). In addition, 9 of the 40 genes associated with DMRs in the hypertrophic cardiomyopathy ontology express myosin heavy chain genes, including 2 that are critical for heart function and development, myosin heavy peptide 6 alpha (*Myh6*) and myosin heavy peptide 7 beta (*Myh7*; Table 4). Analysis of a more specific ontology, cardiac hypertrophy – NF-AT signaling, revealed similar genes as the general hypertrophic cardiomyopathy ontology such as *Myh6*, *Myh7*, *Tnnt2*, *Tnni3*, and *Actc1* (Fig. 7; Table 5). The NF-AT ontology identified transcription factors that were associated with DMRs including GATA binding protein 4 (*Gata4*), myocyte enhancer factor 2c (*Mef2c*), and the transcriptional co-activator calmodulin binding transcription activator 2 (*Camta2*; Table 5). In addition, 9 out of the 28 affected genes in this pathway are guanine nucleotide binding proteins (G-proteins; Table 5).

**Table 4:** Genes in the hypertrophic cardiomyopathy pathway with differentially methylated regions following *in utero* caffeine exposure. (N = 2).

Gene symbol	Accession	Fold change	P-value	Feature
*Myl3*	BC061222	1.72	8.32E-05	Promoter
*Bat5* (HLA-B)	NM_178592	1.63	1.58E-03	Transcript & CpG islands
*Myh14* (MyHC/Myosin II)	NM_028021	1.58	3.47E-05	Promoter
*ANP*	BC089615	1.57	6.76E-04	Promoter
*Cav3* (Caveolin-3)	NM_007617	1.57	8.13E-04	Transcript
*Myh11* (MyHC/Myosin II)	NM_001161775	1.56	2.57E-04	Transcript
*Oxtr* (Galpha(q)-specific peptide GPCRs)	NM_001081147	1.50	9.77E-04	Transcript
*PKC-beta2/cPKC* (conventioanal)	NM_008855	1.49	1.05E-03	Promoter
*Pla2g2a* (PLA2)	NM_001082531	1.48	1.29E-03	Transcript
*Cyp11b2*	BC119321	1.48	3.89E-04	Promoter
*Pla2g5* (PLA2)	NM_001122954	1.47	5.62E-03	Transcript
*Myh7* (MyHC/Myosin II/beta-MHC)	BC121789	1.46	6.31E-04	Transcript
*Mylpf* (MRLC/Myosin II)	NM_016754	1.43	1.17E-03	Promoter
*Vcl* (Vinculin)	BC008520	1.42	1.12E-03	Promoter
*Ndufv2*	BC030946	1.41	1.07E-03	Transcript
*Ppargc1b*	BC150699	1.40	1.74E-04	Promoter
*Myh13* (MyHC/Myosin II)	NM_001081250	1.40	1.66E-03	Promoter
*Vwf* (von Willebrand factor)	NM_011708	1.38	1.29E-03	Transcript
*Myh10* (MyHC/Myosin II)	BC089011	1.37	3.31E-03	Transcript
*PKC*	NM_008860	1.37	9.77E-04	Transcript
*Tnni3* (Troponin I, cardiac)	BC100590	1.35	1.38E-03	Transcript
*Ppargc1* (PGC1-alpha)	NR_027710	1.34	9.33E-04	Transcript
*Myl1* (MELC/Myosin II)	NM_021285	1.31	2.00E-03	Promoter & transcript
*Actb* (Actin)	NM_007393	1.31	2.19E-03	Promoter
*Igf1r*	BC138869	1.30	2.40E-03	Transcript
*Ppm1a* (PP2C)	NM_008910	1.30	4.17E-03	Promoter
*Edn2* (Endothelin-2)	BC037042	1.26	1.48E-03	Promoter
*Actc1* (Actin)	BC062138	1.26	4.07E-03	Promoter
*Myh1* (MyHC/Myosin II)	NM_030679	1.24	9.33E-04	Transcript
*Blat3* (HLA-B)	NM_057171	1.24	1.55E-03	Promoter
*Myl1* (MELC)	NM_021285	1.23	2.14E-03	Transcript
*Myh8* (MyHC/Myosin II)	NM_177369	1.19	1.32E-03	Promoter
*Myoz2*	BC024360	-1.24	2.57E-03	Promoter
*Ace1*	NM_009598	-1.31	1.91E-04	Promoter
*Igfbp1* (IBP1)	BC013345	-1.40	1.05E-03	Promoter
LOC547349 (MHC class I)	NM_001025208	-1.43	3.02E-03	Promoter
*Tnnt2* (Troponin T, cardiac)	NM_001130176	-1.51	5.62E-04	Promoter
*Myh6* (MyHC/Myosin II/alpha-MHC)	NM_010856	-1.52	2.04E-04	Promoter
*Lamp2*	NM_001017959	-1.52	7.59E-04	Promoter
*Mybpc3* (Cardiac MyBP-C)	NM_008653	-1.58	4.17E-03	Promoter
*Tnnc1* (Troponin C, cardiac)	NM_009393	-1.66	1.35E-03	Promoter

**Table 5:** Genes in the cardiac hypertrophy NF-AT signaling pathway with differentially methylated regions following *in utero* caffeine exposure. (N = 2).

Gene symbol	Accession	Fold change	P-value	Feature
*Gng3* (G-protein beta/gamma)	BC029680	1.93	1.51E-03	Promoter
*Ctf1* (Cardiotrophin-1)	NM_007795	1.64	8.13E-04	Promoter
*Gnai3* (G-protein alpha-i family)	NM_010306	1.63	1.86E-03	Promoter
*ANP*	BC089615	1.57	6.76E-04	Promoter
*Camta2*	BC056395	1.57	5.25E-04	Promoter
*Lif*	NM_001039537	1.52	2.69E-04	Transcript
*Gng10* (G-protein beta/gamma)	NM_025277	1.51	1.86E-03	Transcript
*Pik3r2*	NM_008841	1.49	3.39E-05	Transcript
*Gng8* (G-protein beta/gamma)	NM_010320	1.47	8.51E-03	Promoter & transcript
*Myh7* (beta-MHC)	NM_080728	1.46	6.31E-04	Transcript
*Ark* (PKB)	NM_001110208	1.45	1.23E-03	Promoter
*Mef2c*	NM_025282	1.44	2.75E-04/	Promoter
*Gnas* (G-protein alpha-s)	NM_022000	1.43	3.55E-09	Transcript & CpG island
*Shc1*	NM_001113331	1.43	5.37E-04	Transcript
*Adssl1*	NM_007421	1.41	3.55E-03	Promoter
*Map2k5*	BC028260	1.40	1.10E-03	Transcript
*Gng5* (G-protein beta/gamma)	NM_010318	1.37	1.02E-03	Promoter
*Tnni3* (Troponin I, cardiac)	BC100590	1.35	1.38E-03	Promoter & transcript
*Gsk3b*	NM_019827	1.34	2.63E-03	Transcript
*PKC-epsilon*	NM_011104	1.34	5.13E-03	Promoter
*Gata4*	NM_008092	1.32	6.17E-04	Promoter
*Igf1r*	BC138869	1.32	2.40E-03	Promoter & CpG island
*Actc1* (actin)	BC062138	1.26	4.07E-03	Promoter
*Gnb3* (G-protein beta/gamma)	NM_013530	1.25	6.76E-04	Promoter & transcript
*Gngt* (G-protein beta/gamma)	NM_010314	1.23	3.09E-03	Transcript
*Ppp3cb* (Calcineurin A, catalytic)	NM_008914	1.22	2.00E-03	Transcript
*Gng2* (G-protein beta/gamma)	NM_010315	1.18	1.95E-03	Transcript
*Mef2d*	BC011070	1.18	1.74E-03	Transcript
*Gnb11* (G-protein beta/gamma)	NM_001081682	−1.21	7.41E-04	Promoter
*Gngt2* (G-protein beta/gamma)	NM_001038664	−1.43	1.00E-03	Promoter
*Gnaz* (G-protein alpha-i family)	BC014702	−1.43	2.19E-03	Promoter
*Tnnt2* (Troponin T, cardiac)	NM_001130176	−1.51	5.62E-04	Promoter
*Myh6* (alpha-MHC)	NM_010856	−1.52	2.04E-04	Promoter
*Mybpc3* (Cardiac MyBP-C)	NM_008653	−1.58	4.17E-03	Promoter
*MAPK5*	BC100398	−1.59	1.02E-03	Promoter
*Calm3* (Calmodulin)	BC050926	−1.60	2.00E-04	Promoter
*Ppp3cc* (Calcineurin A, catalytic)	BC141079	−1.62	2.19E-04	Promoter
*Pik3cd* (PI3K cat class IA)	NM_001029837	−1.83	2.29E-03	Promoter

To elaborate on the DNA methylation array results, we selected several DMRs at different gene loci related to cardiac hypertrophy including *Mef2c*, *Tnnt2*, *Myh6*, *Myh7*, and *Gata4* and body weight *Ins2* for bisulfite sequencing. Of the six genes examined by BS-sequencing, 3 of them matched the DNA methylation array results including *Mef2c* and *Ins2* which were both hypermethylated and *Myh6* which was hypomethylated in caff+/+ (Table 6). Two genes, *Gata4* and *Myh7*, showed no difference by BS-seq in caff+/+ even though they were both hypermethylated in the DNA methylation array results (Table 6). One gene, *Tnnt2*, was hypomethylated in the caff+/+ group in the array but hypermethylated by BS-seq. (Table 6).

**Table 6:** DNA methylation changes measured by bisulfite sequencing. (N = 2).

Gene name	Gene symbol	Genbank accession	Strand	Distance from TSS	veh+/+methylation %	caff+/+ methylation %	Difference
**Myocyte enhancer factor 2c**	*Mef2c*	NM_025282	+	−764 to −583	75.2%	90.8%	Increased 20.7%
**Insulin II**	*Ins2*	AA986540	-	−699 to −698	68.8%	100.0%	Increased 45.3%
**Insulin II**	*Ins2*	AA986540	-	−275 to −274	79.2%	93.8%	Increased 18.4%
**Troponin T2, Cardiac**	*Tnnt2*	NM_001130176	+	−2494 to −2015	31.8%	55.6%	Increased 74.8%
**Troponin T2, Cardiac**	*Tnnt2*	NM_001130176	+	−1838 to −1496	17.6%	38.3%	Increased 117.6%
**Myosin, heavy polypeptide 6, alpha**	*Myh6*	NM_010856	-	−2095 to −2094	25.0%	4.2%	Decreased 595.2%

TSS: transcriptional start site, caff+/+: caffeine treated A1AR+/+ mice,

veh+/+: 0.09% saline treated A1AR+/+ mice.

**Figure 9:**
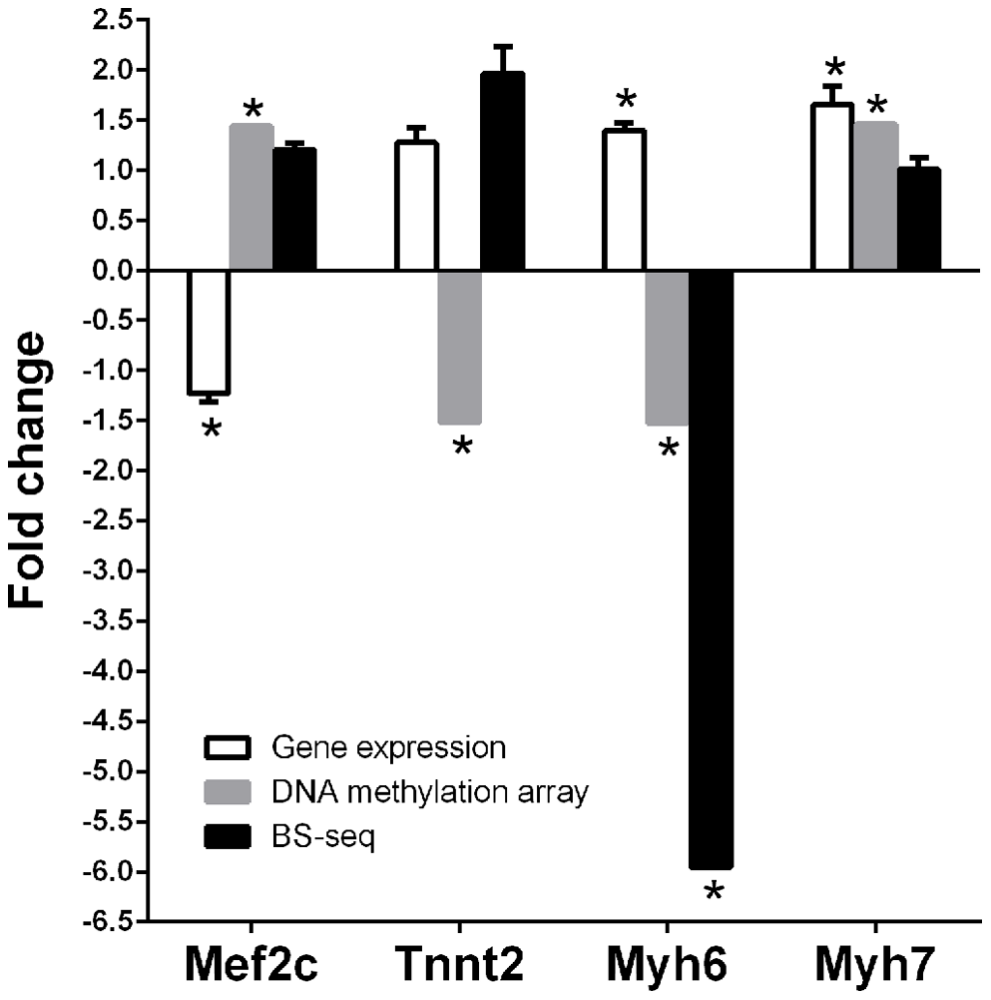
Relation between gene expression and DNA methylation. Expression of critical genes in the cardiac hypertrophy signaling pathway was compared to their DNA methylation status measured by DNA methylation array or bisulfite sequencing. A1AR+/+ mice treated *in utero* with either caffeine or vehicle were compared. Expression or methylation differences are shown as fold-change of caffeine treatments divided by normal saline controls. Gene expression results represent data from three repeats of qPCR measurements (N = 3 per group, genomic DNA and mRNA were extracted from left ventricles of the same animals). Student's t-test used and error bars are SEM. * indicates P≤0.05.

To determine if the DNA methylation changes observed between veh+/+ and caff+/+ affect gene expression, we performed quantitative real-time PCR. We examined the gene expression of *Mef2c*, *Tnnt2*, *Myh6*, and *Myh7* in adult left ventricles. Hypermethylation of DNA is generally associated with a decrease in gene expression and this was observed for *Mef2c* but not *Myh7*, indicating that DNA methylation changes seen for *Myh7* does not affect expression (Fig. 9). Hypomethylation of DNA is generally associated with an increase in gene expression, and that was observed for *Myh6* (Fig. 9).

Analysis of DMRs associated with miRNA sites and promoters identified many pathways and genes related to cardiovascular biology. Using the IPA software, several Cardiotoxicity Pathways were identified including cardiac inflammation, cardiac dilation, and cardiac infarction (Table 7). Analysis of the miRNA regions identified 103 regions that were significantly differentially methylated (Table 8). Of the miRNA promoter regions with DMRs, two were related to cardiac hypertrophy including miR-208b and miR-499 (Table 8) [49], [50]. These miRNAs are located within introns of the *Myh7* and *Myh7b* genes. MiR-208b is located within intron 31 of the *Myh7* gene which is also differentially methylated and miR-499 is located in intron 19 of *Myh7b*. However, unlike *Myh7* which was hypermethylated, regions within the promoter for both miR-208b and miR499 were hypomethylated.

**Table 7:** Significantly enriched miRNA pathways from Ingenuity Pathway Analysis following *in utero* caffeine exposure. (N = 2).

	Top pathways	p-value	# of changed genes
**Diseases and disorders**	Reproductive system disease	9.04E-17	18
	Cancer	1.61E-14	26
	Hematological disease	1.97E-12	10
	Endocrine system disorders	1.27E-11	16
	Inflammatory disease	1.03E-09	11
**Physiological development**	Connective tissue development and function	1.39E-03	4
	Nervous system development and function	2.22E-03	1
	Respiratory system development and function	2.22E-03	1
	Tissue development	2.22E-03	3
	Tumor morphology	2.22E-03	2
**Cardiotoxicity**	Cardiac inflammation	4.78E-06	2
	Congenital heart anomaly	2.60E-04	2
	Cardiac dilation	4.14E-02	1
	Cardiac infarction	4.51E-02	2
	Pulmonary hypertension	4.78E-02	1

**Table 8:** miRNAs with differentially methylated regions in their promoters, ±1.35 fold change cutoff. (N = 2).

Accession	ID	Fold change	P-value
MI0009955	mmu-mir-1958	2.09	6.61E-04
MI0004636	mmu-mir-497	1.76	1.02E-04
MI0000237	mmu-mir-195	1.76	1.02E-04
MI0006295	mmu-mir-466j	1.76	1.62E-03
MI0005515	mmu-mir-504	1.76	3.89E-03
MI0000817	mmu-mir-335	1.73	6.31E-03
MI0000730	mmu-mir-7b	1.66	6.76E-04
MI0000233	mmu-mir-191	1.63	8.91E-04
MI0001447	mmu-mir-425	1.63	8.91E-04
MI0009948	mmu-mir-1953	1.62	4.57E-05
MI0000394	mmu-mir-296	1.62	5.75E-04
MI0000398	mmu-mir-298	1.62	5.75E-04
MI0000584	mmu-mir-34a	1.56	3.31E-03
MI0009936	mmu-mir-1946a	1.55	2.24E-03
MI0000595	mmu-mir-324	1.54	3.63E-03
MI0009931	mmu-mir-1942	1.52	4.79E-04
MI0004652	mmu-mir-687	1.52	1.23E-03
MI0000570	mmu-mir-22	1.48	2.63E-04
MI0001165	mmu-mir-370	1.47	2.63E-04
MI0005496	mmu-mir-421	1.45	6.76E-04
MI0004125	mmu-mir-374	1.45	6.76E-04
MI0004123	mmu-mir-675	1.44	3.16E-06
MI0001146	mmu-mir-384	1.44	1.41E-03
MI0000691	mmu-mir-32	1.43	6.76E-04
MI0000702	mmu-mir-219-1	1.43	4.37E-03
MI0005482	mmu-mir-147	1.42	1.66E-03
MI0005521	mmu-mir-92b	1.41	2.04E-03
MI0000250	mmu-mir-207	1.41	7.94E-03
MI0009967	mmu-mir-1946b	1.39	4.57E-04
MI0000725	mmu-mir-125b-1	1.36	9.55E-04
MI0004258	mmu-mir-672	1.36	2.75E-03
MI0005552	mmu-mir-208b	-1.36	1.00E-03
MI0010752	mmu-mir-2139	-1.36	1.62E-03
MI0000171	mmu-mir-149	-1.40	1.05E-03
MI0000227	mmu-mir-185	-1.40	2.24E-03
MI0009963	mmu-mir-1966	-1.43	1.41E-04
MI0004676	mmu-mir-499	-1.43	2.82E-03
MI0000799	mmu-mir-382	-1.44	2.04E-04
MI0000160	mmu-mir-134	-1.44	2.04E-04
MI0004134	mmu-mir-668	-1.44	2.04E-04
MI0003492	mmu-mir-485	-1.44	2.04E-04
MI0005497	mmu-mir-453	-1.44	2.04E-04
MI0000176	mmu-mir-154	-1.44	2.04E-04
MI0004589	mmu-mir-496	-1.44	2.04E-04
MI0000730	mmu-mir-7b	-1.44	4.47E-03
MI0000583	mmu-mir-96	-1.46	5.13E-04
MI0000225	mmu-mir-183	-1.46	5.13E-04
MI0005475	mmu-mir-882	-1.47	2.40E-03
MI0003484	mmu-mir-483	-1.48	1.29E-04
MI0008315	mmu-mir-1905	-1.52	7.08E-04
MI0004708	mmu-mir-721	-1.72	1.17E-03
MI0009918	mmu-mir-1929	-1.84	1.00E-02
MI0004688	mmu-mir-704	-1.91	1.82E-03

### Caffeine induces a decrease in global DNA methylation

**Figure 10:**
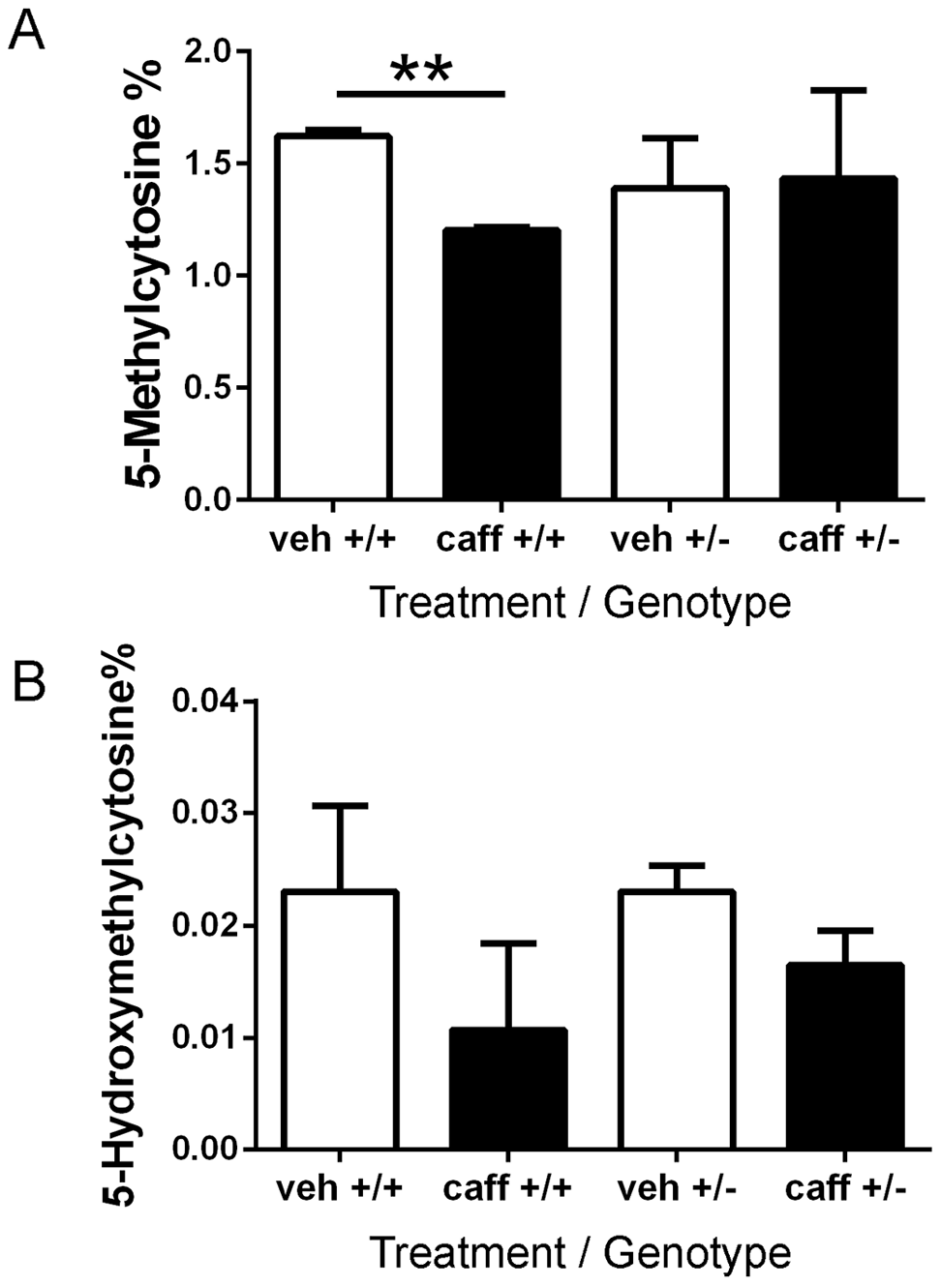
Embryonic caffeine exposure leads to a decrease in global DNA methylation. DNA was isolated from adult left ventricles of mice treated *in utero* with caffeine. (A) The percentage of 5-methylcytosine decreased in caff+/+ hearts compared to veh+/+ controls, but no change in global DNA methylation levels was observed in caff+/− hearts compared to veh+/− hearts. (B) But there was no change in the percentage of 5-hydroxymethylcytosine in the left ventricular DNA in either the caff+/+ or caff+/− hearts. N = 3 per group, each sample was measured 3–4 times, student t-test, ** P = 0.0053).

The DNA methylation array interrogates the portion of the genome that is associated with promoter regions, CpG islands, and miRNA regions. To assess DNA methylation throughout the whole genome, a methylated DNA quantification assay was performed. The whole genome DNA methylation level was compared between veh+/+ and caff+/+ or veh+/− and caff+/− using DNA isolated from adult left ventricles. Caffeine treatment caused a 26% decrease in global DNA methylation in A1AR+/+ hearts, but no change in the level of DNA methylation was detected in the A1AR+/− hearts (Fig. 10). In addition, no significant change in global DNA hydroxymethylation was detected in either A1AR+/+ or A1AR+/− adult hearts following *in utero* caffeine exposure (Fig. 10).

## Discussion

Our previous research demonstrated that A1ARs protect the developing embryo from intra-uterine hypoxia [51], [52]. The loss of A1AR expression in embryos leads to increased embryonic death and severe growth retardation under hypoxic conditions [51], [52]. Further studies demonstrated that hypoxia and/or caffeine treatment, which inhibits A1AR signaling, during embryogenesis had long-lasting effects into adulthood both on cardiac function and body weight [14]. We now demonstrate the importance of normal adenosine signaling through A1ARs during development, as disruption in A1AR action through caffeine treatment leads to increased body weight, reduced cardiac function, and altered cardiac DNA methylation patterns in adulthood.

Previous studies revealed that *in utero* caffeine exposure led to increased percent body fat in adult males but no difference in adult body weight was detected [14]. In the current study, we observed that *in utero* caffeine treatment increased body weight without a change in proportion of body fat. The possible reason for these differences vs. our previous studies may be related to the strain of mice used. The original examination was performed on C57Bl/6, an inbred strain from Charles River Laboratories; the current study examined the A1AR knockout line that is on a mixed background of 129/OlaHsd/C57Bl/6. The difference that we observe in metabolic function between the different mouse strains may be due to differences in DNA methylation changes in response to caffeine treatment. Further experiments into the strain differences in weight and body fat results will need to be performed, especially on an outbred strain. In both strains examined, it is important to note that early embryonic exposure to caffeine induced long-term effects in adult mice.

As with previous studies [14], this study demonstrated altered cardiac function, including decreased cardiac output. Previous reports indicated that *in utero* caffeine exposure caused a decrease in fractional shortening [14]. In addition, this study identified changes in heart morphology, including increased wall thickness following *in utero* caffeine treatment. Previous research showed that caffeine treatment affected the size of embryonic hearts [14]; in this study we observed increased ventricular wall thickness in adult hearts following early caffeine exposure. The increased ventricular wall thickness resulted in reduced ventricular volume and reduced cardiac output. The increased wall thickness and left ventricle mass that we observed are consistent with cardiac concentric hypertrophy, which is characterized by an increase in cardiac wall thickness with a reduced chamber volume. These results suggest that *in utero* caffeine exposure affects cardiac development, which leads to concentric hypertrophy in adulthood to compensate for reduced function. Concentric hypertrophy can eventually be maladaptive when stroke volumes are reduced and diastolic function is compromised.

Altered DNA methylation represents a potential mechanism for translating *in utero* exposure to caffeine into the phenotypic changes observed in adult mice, including increased body weight and cardiac hypertrophy. The developing embryo and heart are sensitive to factors that can alter DNA methylation at early embryonic stages including E8.5 [40], the stage at which we treated pregnant dams with caffeine. DNA demethylation and *de novo* DNA methylation are actively and passively occurring during early embryonic stages. After fertilization, paternal DNA is rapidly demethylated and maternal DNA is passively demethylated until implantation, when *de novo* DNA methylation increases between E3.5 to E10.5 in mice [40]. This period is critical for re-establishing DNA methylation patterns; therefore factors that affect methylation during this time window could have long lasting effects.

The ability of caffeine to alter DNA methylation and gene expression has been demonstrated for the steroidogenic acute regulatory protein (*StAR*) gene in adrenocortical cells [53]. A change in *StAR* expression was attributed to the demethylation of a single CpG site in the *StAR* promoter following caffeine treatment [53]. Our analysis demonstrated that caffeine causes both a genome-wide decrease in methylation, as well as a large number of hypermethylated regions. The induction of both hyper- and hypo-methylated DNA regions in the genome by caffeine was also observed in cultured rat hippocampal neurons [15]. Although these data may seem contradictory, a global decrease and regional increase in DNA methylation has been observed in other biological systems including cancer [54], [55]. In addition, caffeine exposure decreased promoter methylation in L6 rat myotubes that was paralleled by an increase in the respective gene expression [56]. These observations and our data may indicate that changes in DNA methylation associated with caffeine treatment may be the result of more than one pathway. For example, caffeine may affect the activity or expression of both DNA methylation enzymes (DNMTs) and demethylation agents (Tets). The altered activity of these enzymes could then lead to the altered DNA methylation patterns we and others observe. The genes affected by altered DNA methylation may be dependent on the tissue and the timing of treatment. For example, caffeine treatment at E8.5 leads to altered DNA methylation and altered expression of genes associated with cardiac hypertrophy in the adult heart.

Several cardiac hypertrophic pathways were identified with altered DNA methylation patterns following caffeine treatment. The cardiac hypertrophy pathway was identified within multiple databases. Because the cardiac hypertrophy pathways identified by ontology were consistent with the phenotype observed in adult offspring of pregnant dams treated with caffeine, we further analyzed the differentially methylated genes within these pathways. Although we discovered many genes associated with cardiac hypertrophy that displayed altered DNA methylation patterns, not all of these changes will necessarily affect gene expression. To initially analyze the functional effects of altered DNA methylation on gene expression, we performed bisulfite sequencing to identify which DNA methylation sites were changed. Next, we performed real-time PCR to examine the expression level of genes associated with differentially methylated regions.

The first set of genes analyzed were part of the “*NF-AT* signaling in cardiac hypertrophy” pathway. Cardiac hypertrophy is mediated by three main transcription factors *Mef2*, *NF-AT*, and *Gata4* [57], and all of these genes were linked with changes in DNA methylation in our analysis. We analyzed two of these genes further (*Mef2c* and *Gata4*) and demonstrated that an increase in methylation in the promoter region of *Mef2c* correlates with a decrease in *Mef2c* gene expression in the adult heart. Further analysis of *NF-AT3* and *Mef2d*, which both show altered DNA methylation patterns and are important factors during cardiac hypertrophy, is also warranted [57].

We also analyzed myosin heavy chain alpha (*Myh6*) and myosin heavy chain beta (*Myh7*), as their expression is altered during cardiac hypertrophy [50], [57]. During development *Myh6* and *Myh7* are expressed differently with *Myh7* as the predominant fetal isoform and *Myh6* as the dominate form in adults mice [58]. *Myh7* is still expressed in adulthood but an increase in its expression during adulthood is a common feature of cardiac hypertrophy [59]. Our analysis revealed that both *Myh6* and *Myh7* were up-regulated at the level of gene expression. The increase in *Myh6* expression was consistent with a decrease in DNA methylation in its promoter region. However, DNA methylation analysis by both BS-seq and methylation array indicated an increase in DNA methylation in the *Myh7* transcript region. This apparent disconnect between DNA methylation status and gene expression level for *Myh7* may be explained in part by the fact that the promoter region for miR-208b was hypomethylated. MiR-208b is encoded within an intron of the *Myh7* gene, such that an increase in miR-208b expression could also result in an increase in *Myh7* expression due to the fact that they are co-regulated [50]. We observed by real-time PCR an up-regulation of *Myh7* gene expression which is consistent with the phenotype of cardiac hypertrophy observed. Future analysis will focus on elucidating the mechanism by which caffeine exposure causes changes in *Myh6* and *Myh7* methylation, and identifying the specific methylation sites that are important for regulating their expression.

The changes observed with caffeine treatment were seen only when A1ARs were expressed. Loss of A1ARs in the knockout mice (A1AR−/−) protected the embryos and adults from effects of *in utero* caffeine exposure. These findings indicate that A1ARs mediate the effects of caffeine on the developing embryo that lead to long-term changes in cardiac function and body weight as well as long-term changes in DNA methylation patterns. Although we observed changes in DNA methylation in response to caffeine in the absence of A1AR expression, there were fewer differences and we did not observe effects on cardiac function or body weight in these treated mice. These data indicate that caffeine has specific effects on DNA methylation by acting through A1ARs and producing a specific phenotype. In addition, these results suggest that embryonic and cardiac developments are sensitive to changes in A1AR action.

Some of the limitations of this study include the number of animals examined and using i.p. injection as the route of caffeine administration, which is not the normal way of ingesting caffeine in humans. Many parameters, including route of administration, number of exposures including chronic exposure and peak serum levels, need to be considered before commenting on the risk of caffeine exposure in humans [60]. In our previous study, we did not see differences in adult female percent body fat with *in utero* caffeine treatment, which is one reason female mice were not examined in this study [14]. Further studies on the effect of *in utero* caffeine on females will need to be performed in order to overcome this limitation. Caffeine has also been shown to have beneficial effects in neurological and immunological disorders_ENREF_59 [61]–[63]. Thus further clinical and animal studies are needed to assess the effectiveness and safety of caffeine treatment during human pregnancy before recommendations can be made.

This report begins to answer an important question: does *in utero* caffeine exposure increase the susceptibility of an individual developing cardiac or metabolic disease in adulthood? This study is an initial step that indicates that *in utero* caffeine exposure can have long lasting effects into adulthood and that caffeine can alter the DNA methylation pattern in the heart during early stages of embryonic development.
