# Correction: Embryonic Caffeine Exposure Acts via A1 Adenosine Receptors to Alter Adult Cardiac Function and DNA Methylation in Mice

**DOI:** 10.1371/journal.pone.0097212

**Published:** 2014-05-02

**Authors:** The PLOS ONE Staff

Table 3 is incorrect. The authors have provided the correct version of Table 3 here.

**Table 3:** Significantly enriched gene pathways from Ingenuity Pathway Analysis

	Top pathways	p-value	# of changed genes
**Diseases and disorders**	Neurological diseases	1.29E-05	591
	Cancer	3.76E-05	455
	Developmental disorder	4.21E-05	500
	Cardiovascular disease	5.59E-05	440
	Nutritional disease	9.32E-05	182
**Physiological development**	Organismal development	3.53E-06	993
	Organismal survival	4.73E-06	621
	Embryonic development	3.47E-05	632
	Organ development	3.47E-05	557
	Skeletal and muscular system development and function	3.47E-05	393
**Top toxicity lists**	Cardiac hypertrophy	6.41E-05	114/323
	Renal necrosis/cell death	3.56E-04	128/383
	Cardiac fibrosis	4.71E-03	58/166
	Mechanism of gene regulation by peroxisome proliferators via PPARa	1.02E-02	35/95
	Liver proliferation	2.38E-02	61/189
**Cardiotoxicity**	Cardiac hypertrophy	5.59E-05	115
	Pulmonary hypertension	6.60E-04	22
	Cardiac fibrosis	2.43E-03	58
	Cardiac arrhythmia	1.44E-02	53
	Cardiac output	1.97E-02	15
